# Supplementary material for: The Small RNA Universe of Capitella teleta
Source: Front Mol Biosci. 2022 Feb 25;9:802814. doi: 10.3389/fmolb.2022.802814 (PMC8915122; doi:10.3389/fmolb.2022.802814)
Supplement: Supplementary file 1 [file DataSheet1.ZIP › Supplement/known/CAPTEscaffold_114_8281.pdf]

Provisional ID	:	CAPTEscaffold_114_8281
Score total	:	40928.1
Score for star read(s)	:	3.9
Score for read counts	:	40917.7
Score for mfe	:	1.9
Score for randfold	:	1.6
Score for cons. seed	:	3
Total read count	:	80270
Mature read count	:	68702
Loop read count	:	0
Star read count	:	11568

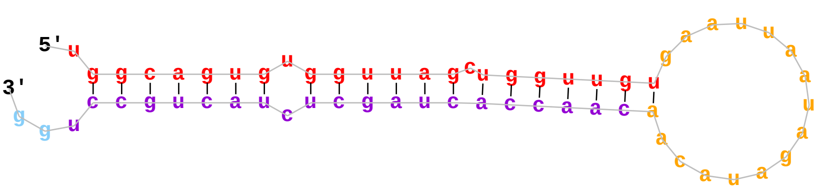

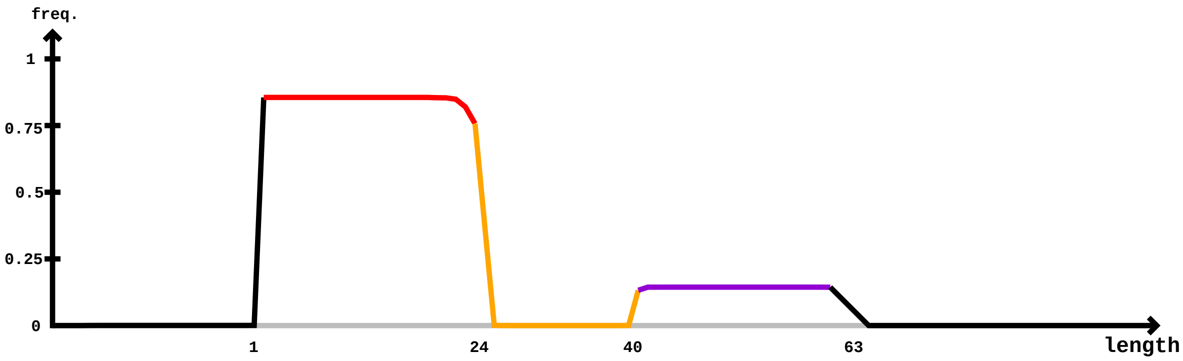


```
                         Mature                              Star
5'- agccacugcuuguguuucgcuggcagugugguuagcugguugugaauuaauagauacaacaaccacuagcucuacugccugggaaauacaaaauuaugucagccucucugguu  -3'  obs
    agccacugcuuguguuucgcuggcagugugguuagcugguugugaauuaauagauacaacaaccacuagcucuacugccugggaaauacaaaauuaugucagccucucugguu       exp
    agccacugcuuguguuucgcuggcagugugguuagcugguugugaauuaauagauacaacaaccacuagcucuacugccugggaaauacaaaauuaugucagccucucugguu       known
    .........((((((((.(.(((((((.((((((.(((((((.................))))))))))))).))))))).).))))))))).........((((.....))))   reads  mm  sample
    agccacugcuuguguuucgc.............................................................................................   1      0   seq
    .gccacugcuuguguuucgc.............................................................................................   1      0   seq
    .gccacugcuuguguuucgcug...........................................................................................   1      0   seq
    ..ccacugcuuguguuucgc.............................................................................................   14     0   seq
    ..ccacAgcuuguguuucgc.............................................................................................   1      1   seq
    ..ccacugcuuguguuucgcug...........................................................................................   5      0   seq
    ..ccacugcuuguguuucAcug...........................................................................................   1      1   seq
    ...cacugcuugAguuucgcug...........................................................................................   1      1   seq
    ...cacugcuuguguuucgcug...........................................................................................   5      0   seq
    ..................gcuggcagugugguuagcugguu........................................................................   1      0   seq
    ..................gcuggcagugugguuagcugguug.......................................................................   1      0   seq
    ..................Ucuggcagugugguuagcugguugu......................................................................   1      1   seq
    ...................cuggcagugugguuagcugguugu......................................................................   10     0   seq
    ....................uggcagugugguuagcug...........................................................................   88     0   seq
    ....................uggcagugugguuagcugg..........................................................................   48     0   seq
    ....................uggcagugugguuagcugA..........................................................................   1      1   seq
    ....................Aggcagugugguuagcuggu.........................................................................   1      1   seq
    ....................uggcagugugguuagcuggG.........................................................................   1      1   seq
    ....................uggcagugugguuagcuggu.........................................................................   412    0   seq
    ....................uggcagAgugguuagcugguu........................................................................   3      1   seq
    ....................uggcaguguAguuagcugguu........................................................................   2      1   seq
    ....................uggcagugugGAuagcugguu........................................................................   3      1   seq
    ....................uggcagugugguuagcuAguu........................................................................   7      1   seq
    ....................uggcaguggguAagcugguu.........................................................................   2      1   seq
    ....................uAgcagugugguuagcugguu........................................................................   4      1   seq
    ....................Gggcagugugguuagcugguu........................................................................   4      1   seq
    ....................ugUcagugugguuagcugguu........................................................................   1      1   seq
    ....................Nggcagugugguuagcugguu........................................................................   2      1   seq
    ....................uggGagugugguuagcugguu........................................................................   2      1   seq
    ....................uNgcagugugguuagcugguu........................................................................   1      1   seq
    ....................uggcagugugAuuagcugguu........................................................................   1      1   seq
    ....................uggcagugugguuagcugguu........................................................................   2274   0   seq
```

```
                    Mature                                  Star
agccacugcuuguguuucgcuggcagugugguuagcugguugugaauuaauagauacaacaaccacuagcucuacugccugggaaauacaaaauuaugucagccucucugguu
....................uggcagugugguuagcCgguu........................................................................	2	1	seq
....................uggcaguAugguuagcugguu........................................................................	1	1	seq
....................uggcagugugguuGgcugguu........................................................................	1	1	seq
....................uggcagugugguuagGugguu........................................................................	1	1	seq
....................uggcagCgugguuagcugguu........................................................................	1	1	seq
....................Aggcagugugguuagcugguu........................................................................	5	1	seq
....................uggcagugugguuagcugguGg.......................................................................	9	1	seq
....................uggcaguAugguuagcugguug.......................................................................	2	1	seq
....................ugUcagugugguuagcugguug.......................................................................	1	1	seq
....................uggAagugugguuagcugguug.......................................................................	1	1	seq
....................Gggcagugugguuagcugguug.......................................................................	7	1	seq
....................uggcagugugguuagcuggAug.......................................................................	1	1	seq
....................uggcagugugguAuagcugguug......................................................................	3	1	seq
....................uAgcagugugguuagcugguug.......................................................................	3	1	seq
....................uggcagugugguuagcuggCug.......................................................................	2	1	seq
....................uggcagugugguuaAcugguug.......................................................................	2	1	seq
....................uggcagugugguuagcuAguug.......................................................................	11	1	seq
....................uggcagugugguuUgcugguug.......................................................................	2	1	seq
....................uggcagugugguuagcugAuug.......................................................................	1	1	seq
....................uggGagugugguuagcugguug.......................................................................	3	1	seq
....................uggcagugugguuagcugguug.......................................................................	4722	0	seq
....................uggUagugugguuagcugguug.......................................................................	1	1	seq
....................uggcagugugguuagcugguuU.......................................................................	205	1	seq
....................uggcagugugguuaCcugguug.......................................................................	1	1	seq
....................Aggcagugugguuagcugguug.......................................................................	11	1	seq
....................uggcaguguggGagcugguug.......................................................................	1	1	seq
....................uggcaguguAguuagcugguug.......................................................................	9	1	seq
....................uggcagugugguuagcCgguug.......................................................................	1	1	seq
....................uggcagugAgguuagcugguug.......................................................................	3	1	seq
....................uggcagugugguuagcugguuA.......................................................................	3	1	seq
....................uggcaguguggCagcugguug........................................................................	1	1	seq
....................uggcagAgugguuagcugguug.......................................................................	6	1	seq
....................uggcagugugguuagcAgguug.......................................................................	1	1	seq
....................uggcagugugguuagcuUguug.......................................................................	1	1	seq
....................Cggcagugugguuagcugguug.......................................................................	1	1	seq
....................uggcagugCgguuagcugguug.......................................................................	2	1	seq
....................uggcGgugugguuagcugguug.......................................................................	3	1	seq
....................uggcaguguUguuagcugguugu......................................................................	5	1	seq
....................uggcagugugguuaCcugguugu......................................................................	1	1	seq
....................uggcGgugugguuagcugguugu......................................................................	6	1	seq
....................uggcagugugguuagcuggCugu......................................................................	8	1	seq
....................uggcagugugguuagcuggGugu......................................................................	10	1	seq
....................uggcagugugguuagcugguAgu......................................................................	24	1	seq
....................uggcagugugguuagGugguugu......................................................................	5	1	seq
....................uggcagCgugguuagcugguugu......................................................................	7	1	seq
....................Nggcagugugguuagcugguugu......................................................................	21	1	seq
....................uggcCgugugguuagcugguugu......................................................................	2	1	seq
....................uggcagugugguuagcugguCgu......................................................................	8	1	seq
....................uggcagAgugguuagcugguugu......................................................................	39	1	seq
....................uggcagugugguuagAugguugu......................................................................	10	1	seq
....................uggcaguUugguuagcugguugu......................................................................	7	1	seq
....................uggcUgugugguuagcugguugu......................................................................	3	1	seq
....................uggcagGgugguuagcugguugu......................................................................	5	1	seq
....................uggcagugugguuagcugguugG......................................................................	41	1	seq
....................uggcaguguggGuagcugguugu......................................................................	4	1	seq
....................uggcaguguggCagcugguugu.......................................................................	10	1	seq
....................ugCcagugugguuagcugguugu......................................................................	9	1	seq
....................Gggcagugugguuagcugguugu......................................................................	65	1	seq
....................uggcagugugguuagcugguGgu......................................................................	3	1	seq
....................uggcagugugUuuagcugguugu......................................................................	7	1	seq
....................uggcaAugugguuagcugguugu......................................................................	8	1	seq
....................uggcaguCugguuagcugguugu......................................................................	5	1	seq
....................uggcagugugguuagcAgguugu......................................................................	15	1	seq
....................uggcagugugguuagcugguuUu......................................................................	31	1	seq
....................Aggcagugugguuagcugguugu......................................................................	138	1	seq
....................uggUagugugguuagcugguugu......................................................................	8	1	seq
....................uggcagugugguuaAcugguugu......................................................................	4	1	seq
....................uggcagugugguuagcuUguugu......................................................................	2	1	seq
....................uggcagugGgguuagcugguugu......................................................................	1	1	seq
....................uggcaUugugguuagcugguugu......................................................................	3	1	seq
```

Mature **Star**

```
agccacugcuuguguuucgcuggcagugugguuagcugguugugaauuaauagauacaacaaccacuagcucuacugccugggaaauacaaaauuaugucagccucucugguu
....................uggcagugugguuagcugUuugu......................................................................  5      1  seq
....................uggcagugugAuuagcugguugu......................................................................  5      1  seq
....................uggcagugugguuGgcugguugu......................................................................  9      1  seq
....................uggcagugugCuuagcugguugu......................................................................  3      1  seq
....................uAgcagugugguuagcugguugu......................................................................  49     1  seq
....................uggcagugugguuagcugguugC......................................................................  5      1  seq
....................ugUcagugugguuagcugguugu......................................................................  8      1  seq
....................uUgcagugugguuagcugguugu......................................................................  5      1  seq
....................uggcaguguAguuagcugguugu......................................................................  76     1  seq
....................uggcagugugguuagcugAuugu......................................................................  10     1  seq
....................uCgcagugugguuagcugguugu......................................................................  9      1  seq
....................uggcagugugguuagcugguuAu......................................................................  15     1  seq
....................uggcagugugguuagcuCguugu......................................................................  4      1  seq
....................uggcagugugguuaUcugguugu......................................................................  2      1  seq
....................uggcagugugguuagcuggAugu......................................................................  18     1  seq
....................uggcagugugguuagcCgguugu......................................................................  8      1  seq
....................uggcaguguggAuagcugguugu......................................................................  26     1  seq
....................uggcagugugguGagcugguugu......................................................................  3      1  seq
....................uNgcagugugguuagcugguugu......................................................................  2      1  seq
....................uggAagugugguuagcugguugu......................................................................  12     1  seq
....................Cggcagugugguuagcugguugu......................................................................  7      1  seq
....................uggcaCugugguuagcugguugu......................................................................  1      1  seq
....................ugAcagugugguuagcugguugu......................................................................  13     1  seq
....................uggcagugugguuagcGgguugu......................................................................  2      1  seq
....................uggcagugugguuagcugguugA......................................................................  89     1  seq
....................uggGagugugguuagcugguugu......................................................................  2      1  seq
....................uggcaguguCguuagcugguugu......................................................................  2      1  seq
....................uggcagugugguAagcugguugu......................................................................  9      1  seq
....................uggcaguAugguuagcugguugu......................................................................  7      1  seq
....................uggcagugugguuagcugguuCu......................................................................  3      1  seq
....................uggcagugAgguuagcugguugu......................................................................  16     1  seq
....................uggcaguguggCuagcugguugu......................................................................  11     1  seq
....................uggcagugCgguuagcugguugu......................................................................  5      1  seq
....................uggcagugugguuagcuAguugu......................................................................  84     1  seq
....................uggcagugugguuagUugguugu......................................................................  14     1  seq
....................uggcagugugguuUgcugguugu......................................................................  3      1  seq
....................uggcagugugguuagcugCuugu......................................................................  5      1  seq
....................uggcagugugguuagcugguugu......................................................................  58325  0  seq
....................uggcagugugguuagcugguuguA.....................................................................  156    1  seq
....................uggcagugugguuagcugguuguU.....................................................................  1130   1  seq
....................uggcagugugguuagcugguugug.....................................................................  27     0  seq
....................uggcagugugguuagcugguuguUa....................................................................  13     1  seq
....................uggcagugugguuagcugguugugU....................................................................  7      1  seq
....................uggcagugugguuagcugguuguAa....................................................................  40     1  seq
....................uggcagugugguuagcugguugugaU...................................................................  2      1  seq
....................uggcagugugguuagcugguuguAaa...................................................................  7      1  seq
....................uggcagugugguuagcugguuguUaa...................................................................  3      1  seq
.....................Ugcagugugguuagcuggu.........................................................................  1      1  seq
.....................ggcagugugguuagcugguug.......................................................................  1      0  seq
.....................ggcagugugguuagcugguugu......................................................................  2      0  seq
.....................Ugcagugugguuagcugguugu......................................................................  5      1  seq
.....................ggcagugugguuagcugguuUu......................................................................  1      1  seq
.....................gAcagugugguuagcugguugu......................................................................  1      1  seq
......................gcagugugguuagcugguug.......................................................................  1      0  seq
......................gcagugugguuagcugguugu......................................................................  8      0  seq
......................gcagugugguuagcugguuguU.....................................................................  1      1  seq
......................gcagugugguuagcugguugug.....................................................................  1      0  seq
........................cagugugguuagcugguugu.....................................................................  2      0  seq
.........................agugugguuagcugguugu.....................................................................  1      0  seq
..........................gugugguuagcugguugu.....................................................................  3      0  seq
..........................gugugguuagcugguuguUa...................................................................  1      1  seq
........................................................caacaaccacuagcucuacugccu.................................  1      0  seq
.........................................................aacaaccacuagcucuacugccu.................................  1      0  seq
..........................................................Ccaaccacuagcucuacugccu.................................  10     1  seq
..........................................................acaaccacuagcucuacugccu.................................  9      0  seq
...........................................................caaccacuagcucuacug....................................  1      0  seq
...........................................................caaccacuagcucuacugc...................................  2      0  seq
...........................................................caaccacuagcucuacugcc..................................  25     0  seq
...........................................................caaccacuagcCcuacugccu.................................  2      1  seq
...........................................................caaccacuagcucuaAugccu.................................  2      1  seq
```

Mature Star

```
agccacugcuuguguuucgcuggcagugugguuagcugguugugaauuaauagauacaacaaccacuagcucuacugccugggaaauacaaaauuaugucagccucucugguu
...........................................................caaccacuagcucuacugUcu.................................  1      1  seq
...........................................................caacAacuagcucuacugccu.................................  2      1  seq
...........................................................caaccacuagcucuacugccA.................................  75     1  seq
...........................................................caaccacuagcucAacugccu.................................  4      1  seq
...........................................................caaccacuagcuAuacugccu.................................  9      1  seq
...........................................................caaccacuagcuUuacugccu.................................  4      1  seq
...........................................................caUccacuagcucuacugccu.................................  1      1  seq
...........................................................caaccacuagcucuacugAcu.................................  2      1  seq
...........................................................caaccacuagcGcuacugccu.................................  2      1  seq
...........................................................caaccUcuagcucuacugccu.................................  3      1  seq
...........................................................caaccacuaAcucuacugccu.................................  1      1  seq
...........................................................caaccacuagcucuacuAccu.................................  2      1  seq
...........................................................caaccacuagcucuacugcUu.................................  1      1  seq
...........................................................caaccaGuagcucuacugccu.................................  1      1  seq
...........................................................caaccacuagAucuacugccu.................................  5      1  seq
...........................................................Naaccacuagcucuacugccu.................................  3      1  seq
...........................................................caaccacAagcucuacugccu.................................  6      1  seq
...........................................................caaccacCagcucuacugccu.................................  1      1  seq
...........................................................caaccacuagcucuaGugccu.................................  1      1  seq
...........................................................caaccacuagcucuaUugccu.................................  2      1  seq
...........................................................caacUacuagcucuacugccu.................................  3      1  seq
...........................................................caaccGcuagcucuacugccu.................................  1      1  seq
...........................................................Aaaccacuagcucuacugccu.................................  3      1  seq
...........................................................caaccacuagcucuacugccC.................................  11     1  seq
...........................................................caaccacuGgcucuacugccu.................................  1      1  seq
...........................................................caaccacuagcAcuacugccu.................................  5      1  seq
...........................................................caaccacuagcucuGcugccu.................................  3      1  seq
...........................................................caaccacuagcucuacAgccu.................................  7      1  seq
...........................................................caaccacuaCcucuacugccu.................................  2      1  seq
...........................................................caaccacuagcucuacugcAu.................................  6      1  seq
...........................................................Uaaccacuagcucuacugccu.................................  22     1  seq
...........................................................caaccacuagcucuacugGcu.................................  1      1  seq
...........................................................caaccacuagcucuacugccu.................................  10271  0  seq
...........................................................caaccacuagcucuacCgccu.................................  2      1  seq
...........................................................cGaccacuagcucuacugccu.................................  2      1  seq
...........................................................caaccacuagcucuacuUccu.................................  1      1  seq
...........................................................caaUcacuagcucuacugccu.................................  2      1  seq
...........................................................caaAcacuagcucuacugccu.................................  2      1  seq
...........................................................caaccacuagcucuacugccG.................................  42     1  seq
...........................................................caaccacuagcucuacugccuU................................  28     1  seq
...........................................................caaccacuagcucuacugccug................................  31     0  seq
...........................................................caaccacuagcucuacugccugU...............................  1      1  seq
............................................................aaccacuagcucuacugcc..................................  3      0  seq
............................................................aaccacuagcucuacAgccu.................................  1      1  seq
............................................................aaccacuagcucuacuUccu.................................  1      1  seq
............................................................aaccacuagcucuacugAcu.................................  1      1  seq
............................................................aaccacAagcucuacugccu.................................  1      1  seq
............................................................aaccacuagcucuacugccG.................................  5      1  seq
............................................................aaccacuagcucuacugccA.................................  3      1  seq
............................................................aaccacuagcuAuacugccu.................................  1      1  seq
............................................................aaccUcuagcucuacugccu.................................  2      1  seq
............................................................aaccacuagcucuacugccu.................................  577    0  seq
............................................................aaccacuagcucuacugccug................................  61     0  seq
............................................................aaccacuagcucuacugUcug................................  1      1  seq
............................................................aaccacuagcucuacAgccug................................  1      1  seq
............................................................aaccacuagcucuacugccuU................................  22     1  seq
............................................................aaccacuagcucuacugccugU...............................  8      1  seq
............................................................aaccUcuagcucuacugccugg...............................  1      1  seq
............................................................aaccacuagcucuacugccuAg...............................  2      1  seq
............................................................aaccacuagcucuacugccugg...............................  243    0  seq
............................................................aaccacuagcucuacugcAugg...............................  1      1  seq
............................................................aaccacuagcucuacugccuggA..............................  1      1  seq
............................................................aaccacuagcucuacugccuggU..............................  5      1  seq
..............................................................ccacuagcucuacugccu.................................  2      0  seq
..............................................................ccacuagcucuacugccuggga.............................  1      0  seq
```
